# Supplementary material for: Long-Term Outcomes of the Dietary Approaches to Stop Hypertension (DASH) Intervention in Nonobstructive Coronary Artery Disease: Follow-Up of the DISCO-CT Study
Source: Nutrients. 2025 Aug 6;17(15):2565. doi: 10.3390/nu17152565 (PMC12348568; doi:10.3390/nu17152565)
Supplement: Supplementary file 1 [file nutrients-17-02565-s001.zip › nutrients-3760364-supplementary.pdf]

Table S1. Baseline age vs. 6-year change in selected outcomes (Spearman correlation and age-adjusted linear regression)

Marker	Spearman *r*	*P* value	β-Age (per year)	*P* value
LDL-C, mg/dL	-0.032	0.77	-0.02	0.98
CXCL4, g/mL	-0.13	0.25	-0.09	0.17
RANTES, ng/mL	-0.15	0.18	-0.47	0.26
BMI, kg/m^{-2}	0.16	0.15	0.05	0.08
VFA, cm^2	0.13	0.24	**1.85**	**0.037**
TBF, kg	0.05	0.62	0.18	0.10

β-Age (per year) = effect of one-year increase in baseline age on the 6-year change (Δ = Follow-up – Baseline), estimated from linear regression models adjusted for study allocation (ARMS); BMI, body mass index; LDL-C, low density lipoprotein cholesterol; TBF, total body fat; VFA, visceral fat area. Bold indicates statistical significance at $p < 0.05$.
